# A Novel HDAC6 Inhibitor Enhances the Efficacy of Paclitaxel Against Ovarian Cancer Cells

**DOI:** 10.3390/molecules30132793

**Published:** 2025-06-28

**Authors:** An-Jui Chi, Jui-Ling Hsu, Yun-Xin Xiao, Ji-Wang Chern, Jih-Hwa Guh, Chao-Wu Yu, Lih-Ching Hsu

**Affiliations:** 1 School of Pharmacy, College of Medicine, National Taiwan University, Taipei 10050, Taiwan; a0970697645@gmail.com (A.-J.C.); d97423004@ntu.edu.tw (J.-L.H.); b12403016@ntu.edu.tw (Y.-X.X.); jwchern@ntu.edu.tw (J.-W.C.); jhguh@ntu.edu.tw (J.-H.G.); 2 Department of Nursing, Center for Drug Research and Development, Chang Gung University of Science and Technology, Guishan, Taoyuan 33303, Taiwan

**Keywords:** HDAC6 inhibitor, paclitaxel (Taxol), ovarian cancer, combination therapy, apoptosis

## Abstract

Ovarian cancer cells overexpress HDAC6, and selective HDAC6 inhibitors have been considered potential new drugs for ovarian cancer either alone or in combination with other anticancer agents. We screened 46 potential novel HDAC6 inhibitors in ES-2 ovarian cancer cells and showed that compound **25253** demonstrated the most potent anti-proliferative activity and effective synergy with paclitaxel, which was also validated in TOV21G ovarian cancer cells. The combination of **25253** and paclitaxel significantly induced subG1 and apoptotic cells, revealed by PI staining assay and Annexin V-FITC/PI double staining assay, respectively. Western blot analysis showed downregulation of Bcl-2 and Bcl-XL, and upregulation of Bax and Bak, indicating that apoptosis was mediated through the intrinsic pathway. The combination increased γ-H2AX and p-p53 protein levels, suggesting the induction of DNA damage. Furthermore, HDAC6 was downregulated and acetylated α-tubulin was profoundly increased. Compound **25253** enhanced the inhibitory effect of paclitaxel on cell migration and invasion, possibly due to the extensive accumulation of acetylated α-tubulin, which affected microtubule dynamics. Taken together, the combination of **25253** and paclitaxel synergistically inhibited the growth, migration, and invasion of ovarian cancer cells and induced apoptosis, providing supporting evidence that the combination of HDAC6 inhibitors and paclitaxel may be a promising treatment strategy for ovarian cancer.

## 1. Introduction

Ovarian cancer is the sixth most lethal female cancer in spite of its relatively low incidence, according to the 2025 estimated cancer statistics in the United States [1]. It is also one of the most deadly gynecologic malignancies and ranked eighth both in incidence and mortality in female cancers worldwide based on Globocan 2022 [2]. Ovarian cancer often causes no symptoms at early stages, and it is usually diagnosed at advanced stages, with five-year survival rates ~31% [3]. Surgical debulking followed by chemotherapy, including platinum-based drugs and taxanes, is the standard treatment for advanced ovarian cancer. Other adjuvant therapy options include hormone therapy, targeted therapy such as antiangiogenic therapy and poly (ADP-ribose) polymerase (PARP) inhibitors, and immunotherapy [4,5]. Taxanes such as paclitaxel and docetaxel are commonly used for the treatment of ovarian cancer. Paclitaxel is usually combined with other anticancer drugs to exert better clinical therapeutic outcomes, and many new combination strategies tested in clinical trials indicate better survival outcomes in ovarian cancer [6]. Drug combination provides various advantages in cancer treatment including a lower dose required for each drug, less chance of drug resistance, and the blockade of multiple targets [7].

Human histone deacetylases (HDACs) have been classified into 18 subtypes and grouped into four classes according to their homology to yeast deacetylases. HDACs are associated with tumorigenesis and several pan-HDAC inhibitors have been approved for cancer therapy, such as vorinostat (also known as suberoylanilide hydroxamic acid, SAHA) for cutaneous T cell lymphoma [8]. HDAC6, a unique member of the HDAC family, is classified as class IIb with two functionally independent deacetylase domains and a zinc finger ubiquitin-binding domain; it is predominantly localized to the cytoplasm owing to the presence of a nuclear export sequence and a cytoplasmic anchoring signal SE14 motif, in contrast to other HDACs, which are mainly localized to the nucleus. HDAC6 primarily targets cytoplasmic nonhistone proteins, including α-tubulin, heat shock proteins, cortactin, and certain tumor-associated antigens. Due to its role in regulating nonhistone proteins, HDAC6 is involved in several vital cellular processes including cell motility, cell cycle progression, protein degradation, and protein maturation. Recent studies also revealed that overexpression of HDAC6 correlates with the progression of various cancers, such as ovarian cancer, breast cancer, lung cancer, and prostate cancer. Abnormal expression of HDAC6 can lead to oncogenic cell transformation and tumor metastasis. Several HDAC6 inhibitors have been developed for targeted cancer therapy, and some have entered clinical trials. Thus, HDAC6 is a promising target for the development of novel anticancer drugs [9].

Quinazolinone-, 4-arylquinazolinone, and imidazo [1,2-c]quinazolinone-based derivatives were designed and synthesized as potential HDAC6 inhibitors. Quinazolinone and 4-arylquinazolinone core structures are commonly found in approved drugs with good drug properties and are easy to approach with various functional groups for studying the structure–activity relationship. Imidazo [1,2-c]quinazolinone has a core structure similar to that of tubastatin A, a highly selective HDAC6 inhibitor; additionally, we would like to test novel bulky tricyclic cap groups to obtain HDAC6 selectivity [10]. Among 46 compounds tested, **25253**, **25276**, and **25278** displayed the best growth inhibitory activity against ES-2 ovarian cancer cells. The combination of these compounds with several anticancer drugs was also investigated in a preliminary test, and paclitaxel showed the best combinatorial efficacy. Further studies identified **25253** and paclitaxel as the best combination against ES-2 cells and this combination was also validated in another ovarian cancer cell line, TOV21G. Mechanism studies in ES-2 cells revealed that paclitaxel and **25253** disturbed cell cycle progression, synergistically induced DNA damage, and caused apoptosis via the intrinsic pathway. Taxanes, including paclitaxel, are known to stabilize microtubules and disrupt mitosis [11]. We found that paclitaxel induced acetylated α-tubulin, which may affect microtubule dynamics in ES-2 cells, and **25253** further enhanced this effect, leading to better inhibition of cell migration and invasion.

## 2. Results

### 2.1. Determination of Antiproliferative Effect of 46 Potential Selective HDAC6 Inhibitors in ES-2 Ovarian Cancer Cells

**Figure 1:**
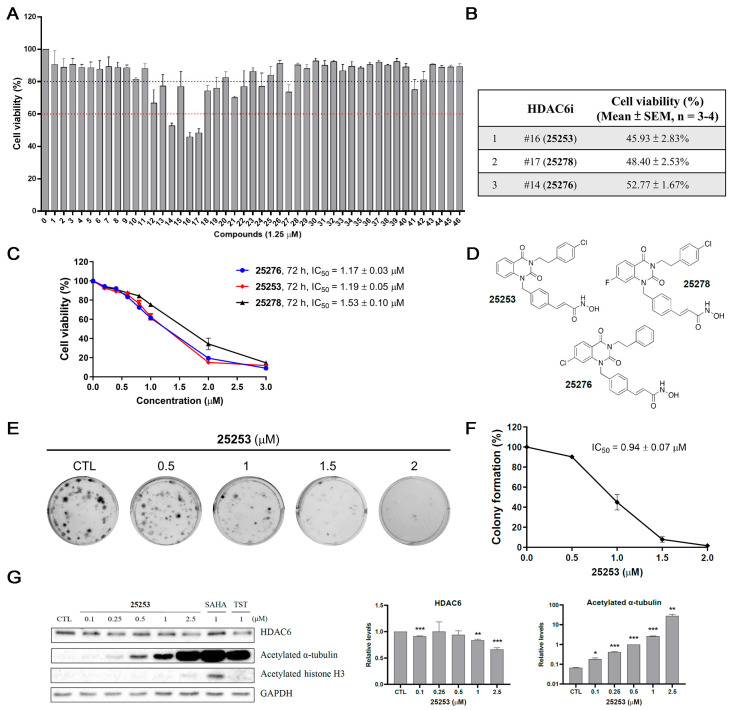
Identification of novel HDAC6 inhibitors against ES-2 ovarian cancer cells. (**A**) Determination of the antiproliferative effect of 46 potential selective HDAC6 inhibitors in ES-2 cells. ES-2 cells were treated with 1.25 μM compounds for 72 h and cell viability was measured by the MTT assay. (**B**) Cell viability of three most potent compounds. HDAC6i: HDAC6 inhibitor. (**C**) Dose–response curves of the three most potent compounds, **25276**, **25253**, and **25278**, in ES-2 cells treated for 72 h. (**D**) Chemical structures of **25253**, **25278**, and **25276**. (**E**) Colony formation assay of **25253** in ES-2 cells. ES-2 cells were treated with indicated concentrations of **25253** for 24 h and then cultured in drug-free medium for 9 days. Colonies were stained with crystal violet. (**F**) Quantitative data of colony formation assay. (**G**) **25253** selectively inhibited HDAC6 determined by Western blot analysis. Data are presented as mean ± SEM of at least three independent experiments. * *p* < 0.05, ** *p* < 0.01, and *** *p* < 0.001 vs. the vehicle control (CTL).

The MTT assay [12] was conducted to determine the cell viability of ES-2 ovarian cancer cells treated with 46 potential selective HDAC6 inhibitors at 1.25 μM for 72 h. Among all compounds tested, #16 (**25253**), #17 (**25278**), and #14 (**25276**) exhibited the most potent anti-proliferative effect with cell viability of 45.93%, 48.40%, 52.77%, respectively (Figure 1A,B). Further dose-dependent experiments revealed that #14 (**25276**), #16 (**25253**) and #17 (**25278**) had 50% inhibitory concentration (IC_50_) values of 1.17 μM, 1.19 μM, and 1.53 μM, respectively, in ES-2 cells treated for 72 h (Figure 1C). These three compounds are quinazolin-2,4-dione derivatives and the chemical structures are shown in Figure 1D.

A colony formation assay was performed to determine the long-term growth inhibitory effect. ES-2 cells were treated with 0–2 μM of **25253** for 24 h and then cultured in a drug-free medium for 9 days. The results showed that **25253** still maintained a dose-dependent growth inhibitory effect after being washed out for 9 days, with an IC_50_ value of 0.94 ± 0.07 μM (Figure 1E,F), indicative of a prolonged antiproliferative effect.

Western blot analysis was conducted to confirm the selectivity of **25253** towards HDAC6. ES-2 cells were treated with 0.1 to 2.5 μM of **25253**, and 1 μM of a well-known pan-HDAC inhibitor SAHA and a selective HDAC6 inhibitor tubastatin A (TST) were included for comparison. As shown in Figure 1G, indeed, **25253** selectively inhibited HDAC6 activity in ES-2 cells as the acetylation of the HDAC6-specific substrate α-tubulin was induced in ES-2 cells treated with as low as 0.1 μM of **25253**, and the acetylated α-tubulin levels were elevated dramatically with increasing concentrations of **25253**. In contrast, acetylation of a nuclear HDAC substrate histone H3 was apparently detected only at 2.5 μM of **25253**, but the level was far below that induced by 1 μM SAHA. Notably, the protein levels of HDAC6 were apparently downregulated by **25253** at 1 and 2.5 μM, indicating that **25253** not only suppressed HDAC6 enzyme activity but also caused downregulation of the enzyme at high concentrations. These data provide evidence that **25253** is a selective HDAC6 inhibitor.

### 2.2. Screening for Anticancer Agents That Can Synergize with HDAC6 Inhibitors Against ES-2 Ovarian Cancer Cells

**Figure 2:**
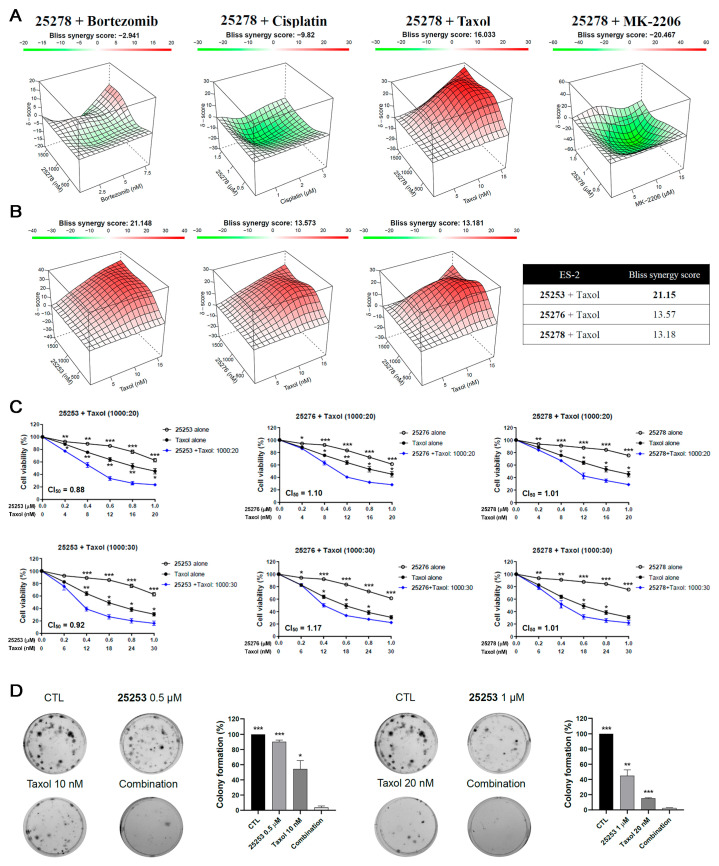
The combination effect of HDAC6 inhibitors and anticancer drugs in ES-2 cells. (**A**) A preliminary test of the combination of **25278** with bortezomib, cisplatin, Taxol, and MK-2206 based on synergy scores calculated by SynergyFinder 3.0. (**B**) The combination of **25253**, **25276**, or **25278** with Taxol showed synergy based on synergy scores. (**C**) The combination of **25253** with Taxol showed the best synergy based on combination index calculated by CompuSyn. ES-2 cells were treated for 72 h and cell viability was determined by the MTT assay. (**D**) Colony formation assay of ES-2 cells treated with **25253** and Taxol either alone or in combination. ES-2 cells were treated with indicated compounds for 24 h and then cultured in drug-free medium for 9 days. Colonies were stained with crystal violet. Data are presented as mean ± SEM of at least three independent experiments except the preliminary test in (**A**). * *p* < 0.05, ** *p* < 0.01, ***, *p* < 0.001 vs. combination.

To develop a potential combination treatment strategy using HDAC6 inhibitors against ovarian cancer, **25278**, one of the most potent compounds, was used for a preliminary test in combination with anticancer agents including paclitaxel (Taxol), bortezomib, cisplatin, and MK-2206 by the MTT assay in ES-2 cells. The combination effect was calculated using SynergyFinder 3.0 software (SynergyFinder: Helsinki, Finland, 2022) with overall effectiveness presented as the synergy score (SC). An SC >10 indicates synergistic activity, between 10 and −10 indicates an additive effect, and below −10 indicates antagonistic activity [13]. As shown in Figure 2A, **25278** combined with bortezomib and cisplatin only had an additive effect (SC of −2.94 and −9.82, respectively) while the combination of **25278** and MK-2206 had a slightly antagonistic effect. Strikingly, combining **25278** with Taxol showed a synergistic effect, with an SC of 16.03. Thus, Taxol was chosen for further combination tests with **25253**, **25276**, and **25278.**

### 2.3. HDAC6 Inhibitor **25253** Displays the Best Synergistic Effect When Combined with Taxol Against ES-2 Cells

To evaluate the combination effect of **25253**, **25276**, and **25278** with Taxol, ES-2 cells were treated with 0.5 μM to 1.5 μM HDAC6 inhibitors and 5 nM to 15 nM Taxol either alone or in combination. As illustrated in Figure 2B, **25253** displayed the strongest synergy, with an SC of 21.15, compared to **25276** and **25278**, with an SC of 13.57 and 13.18, respectively. Thus, **25253**, **25276**, and **25278** all displayed synergy with Taxol according to the SC.

**Figure 3:**
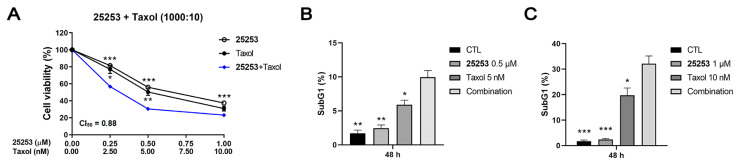
The combination effect of **25253** and Taxol in TOV21G cells. (**A**) The combination of **25253** with Taxol showed synergy in TOV21G cells based on combination index calculated by CompuSyn. Cells were treated for 72 h and cell viability was determined by the MTT assay. (**B**) The combination of 0.5 μM **25253** and 5 nM Taxol synergistically induced cell death in TOV21G cells. (**C**) The combination of 1 μM **25253** and 10 nM Taxol synergistically induced cell death in TOV21G cells. Cells were treated for 48 h and subjected to PI staining and flow cytometric analysis to determine the subG1 population. Quantitative data are presented as mean ± SEM of at least three independent experiments. * *p* < 0.05, ** *p* < 0.01, *** *p* < 0.001 vs. combination.

Based on the 3D map results (Figure 2B), two molar ratios were selected to conduct the constant ratio combination tests and CompuSyn software 1.0 was used to calculate the combination index (CI), an indicator of drug interactions in combination chemotherapy. CI values <1, =1, or >1 indicate synergistic, additive, or antagonistic effects, respectively [14]. In the combination of **25253** and Taxol, both molar ratios (1000:20 and 1000:30) exhibited synergy, with combination index at IC_50_ (CI_50_) values of 0.88 and 0.92, respectively. The IC_50_ values of **25253** and Taxol alone or as combination treatment at a 1000:20 molar ratio are listed in Table S1, Supplementary Materials, and the IC_50_ values of **25253** and Taxol were greatly reduced in the combination treatment. The combination of **25278** and Taxol displayed only an additive effect, while the combination of **25276** and Taxol showed neither a synergistic nor an additive effect at both molar ratios, although all the combination treatments showed better growth inhibitory effects than their respective single treatments (Figure 2C). The synergy between **25253** and Taxol was also confirmed in another ovarian cancer cell line, TOV21G, with a CI_50_ of 0.88 at a molar ratio of 1000:10 (Figure 3A), and the IC_50_ values of **25253** in single and combination treatments were in the submicromolar range (Table S1).

To evaluate the long-term effects of the combination of **25253** and Taxol, the colony formation assay was performed in ES-2 cells. The combination of 0.5 μM **25253** and 10 nM Taxol suppressed colony formation more efficiently compared to either single treatment. Similar results were obtained in the combination of 1 μM **25253** and 20 nM Taxol (Figure 2D). Thus, the combination of **25253** and Taxol not only synergistically reduced cell viability but also suppressed long-term growth of ES-2 cells. The molecular mechanisms underlying the synergy between **25253** and Taxol were further investigated.

### 2.4. Effects of the Combination of **25253** and Taxol on Cell Cycle Progression and Apoptosis in ES-2 Cells

**Figure 4:**
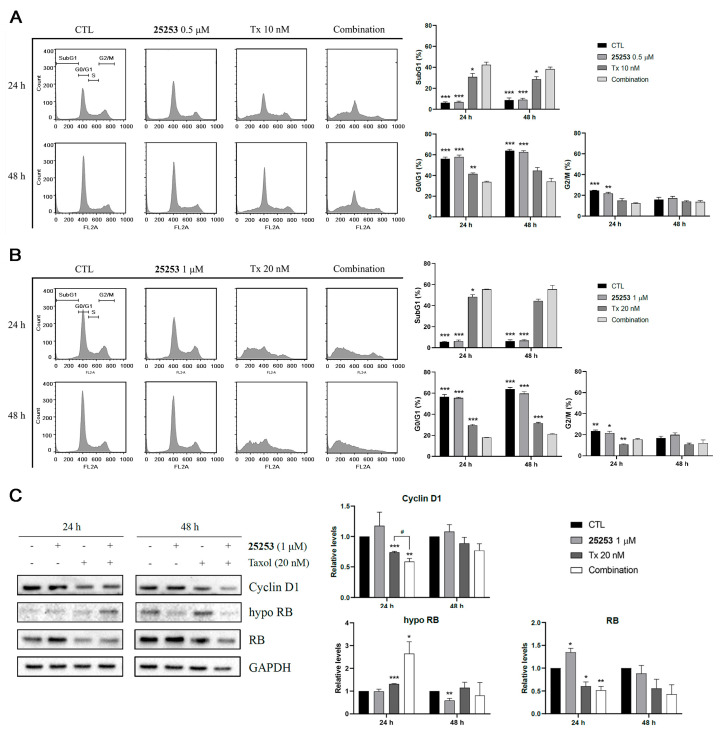
Effects of Taxol combined with **25253** on cell cycle progression and cell cycle-related proteins in ES-2 cells. (**A**) The effect of 0.5 μM **25253** and 10 nM Taxol on cell cycle progression. (**B**) The effect of 1 μM **25253** and 20 nM Taxol on cell cycle progression. Representative histograms of cell cycle distribution are shown on the left and quantitative data are shown on the right. ES-2 cells were treated for 24 h and 48 h and then subjected to PI staining and flow cytometry. * *p* < 0.05, ** *p* < 0.01, *** *p* < 0.001 vs. combination. (**C**) Western blot analysis of cell cycle-related proteins after 24 and 48 h of treatment. * *p* < 0.05, ** *p* < 0.01, *** *p* < 0.001 vs. CTL. ^#^ *p* < 0.05, combination vs. 20 nM Tx. Quantitative data are presented as mean ± SEM of at least three independent experiments.

Propidium iodide (PI) staining followed by flow cytometric analysis was performed to evaluate the effect of the combination of **25253** and Taxol on cell cycle progression in ES-2 cells. As shown in Figure 4A,B, 0.5 or 1 μM **25253** had no apparent effects on cell cycle progression at both 24 and 48 h. In contrast, 10 or 20 nM Taxol (Tx) significantly disrupted cell cycle distribution at 24 h, notably increased the subG1 population (10 nM Tx, *p* < 0.01; 20 nM Tx, *p* < 0.001), and decreased the G0/G1 population (10 nM Tx, *p* < 0.01; 20 nM Tx, *p* < 0.001) compared to the control. These effects persisted after 48 h of treatment. The combination of **25253** and Taxol, both 0.5 μM **25253**/10 nM Taxol and 1 μM **25253**/20 nM Taxol, exhibited significant disruption of the cell cycle with subG1 populations higher than Taxol alone. These subG1 cells were not necessarily all apoptotic cells since it was demonstrated that low concentrations of Taxol prolonged mitosis and caused multipolar divisions, leading to an increase in the subG1 population in which some cells remained viable and non-apoptotic [15,16].

Western blot analysis revealed that both 20 nM Taxol and the combination of 1 μM **25253** and 20 nM Taxol significantly downregulated cyclin D1 relative to the vehicle control, and the combination group showed stronger downregulation compared to Taxol alone (*p* < 0.05) at 24 h with concomitant increases in hypophosphorylated RB levels and decreases in total RB levels, indicative of G0/G1 arrest (Figure 4C), which was the opposite to the data shown in Figure 4A,B with reduced G0/G1 populations obtained from PI staining. These results indicated that under low concentration (20 nM) Taxol treatment, cells were clustered at the subG1 phase, and **25253** further increased this population; Western blot analysis provided evidence supporting the possibility that part of the subG1 cells induced by Taxol or the combination of Taxol and **25253** at 24 h might be small, viable G1 cells exited from prolonged multipolar mitosis rather than apoptotic cells.

**Figure 5:**
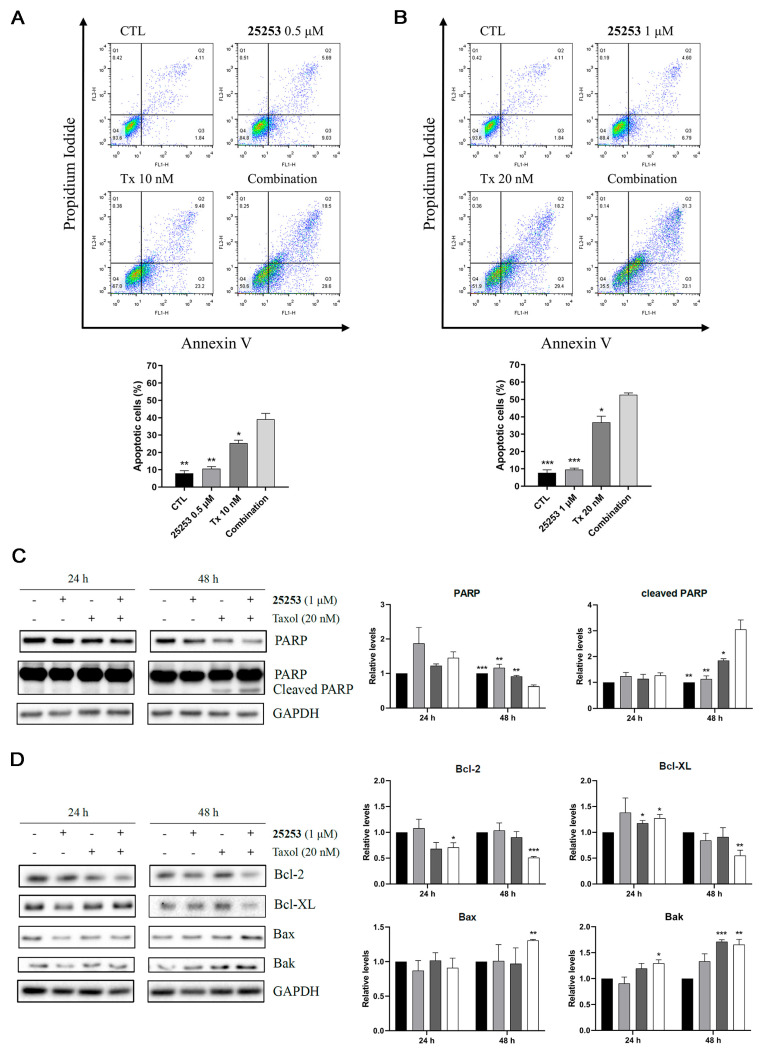
Taxol combined with **25253** induces apoptosis in ES-2 cells. (**A**) Annexin V assay of the combination of 0.5 μM **25253** and 10 nM Taxol. (**B**) Annexin V assay of the combination of 1 μM **25253** and 20 nM Taxol. Representative 2D dot plots and quantitative data are shown. ES-2 cells were treated for 72 h and then harvested for Annexin V assay. Apoptotic cells (%) were calculated as the sum of early (Q3) and late (Q2) apoptotic cells. (**C**) The combination of Taxol and **25253** induced apoptosis after 48 h of treatment. ES-2 cells were treated for 24 h and 48 h and then subjected to Western blot analysis of PARP. * *p* < 0.05, ** *p* < 0.01, *** *p* < 0.001 vs. combination. (**D**) The combination of Taxol and **25253** induced apoptosis via the intrinsic pathway. ES-2 cells were treated for 24 h and 48 h and then subjected to Western blot analysis of Bcl-2 family proteins. * *p* < 0.05, ** *p* < 0.01, *** *p* < 0.001 vs. CTL. Quantitative data are presented as mean ± SEM of at least three independent experiments.

Annexin V-FTIC/PI double staining followed by flow cytometry was then conducted to detect apoptosis in ES-2 cells after 72 h of drug treatment. As illustrated in Figure 5A,B, 0.5 μM or 1 μM **25253** alone did not significantly induce apoptosis (11.14 ± 1.79% or 10.6 ± 0.73% vs. 8.03 ± 1.49% apoptotic cells in the control). However, single treatment with 10 nM or 20 nM Taxol significantly induced apoptotic cells (26.8 ± 2.97%, *p* < 0.01 or 40.17 ± 5.14%, *p* < 0.01 vs. the control). The combination of 0.5 μM **25253** and 10 nM Taxol induced 40.6 ± 4.62% apoptotic cells, and 1 μM **25253** combined with 20 nM Taxol resulted in 56.07 ± 4.23% apoptotic cells, much higher than single treatments.

Western blot analysis of PARP in ES-2 cells treated with 20 nM Taxol alone or in combination with 1 μM **25253** displayed no clear changes at 24 h, but showed a decrease in the pro-form (short exposure time) and an increase in the cleaved form (long exposure time) of PARP at 48 h, indicating that apoptosis was significantly induced at 48 h but not yet at 24 h (Figure 5C). Furthermore, the combination group induced higher levels of cleaved PARP than Taxol alone, which correlated with the results of the Annexin V-FTIC/PI double staining assay (Figure 5B). There were no obvious increases in cleaved PARP at 24 h, further supporting the notion that the subG1 cells induced by Taxol or the combination of Taxol and **25253** at 24 h were viable G1 but not apoptotic cells (Figure 4). The Bcl-2 family proteins play a crucial role in regulating the intrinsic pathway of apoptosis. Our study showed that when 1 μM **25253** was combined with 20 nM Taxol, the pro-survival proteins Bcl-2 and Bcl-XL were downregulated, and the pro-apoptotic proteins Bax and Bak were upregulated at 48 h, suggesting that apoptosis was mediated through the intrinsic pathway (Figure 5D).

The combination of **25253** and Taxol also induced apoptosis in TOV21G cells, revealed by the increases in subG1 populations after 48 h of treatment with 0.5 μM **25253**/5 nM Taxol and 1 μM **25253**/10 nM Taxol, which were more prominent than Taxol alone, as illustrated in Figure 3B,C.

### 2.5. The Combination of **25253** and Taxol Synergistically Induces DNA Damage in ES-2 Cells

**Figure 6:**
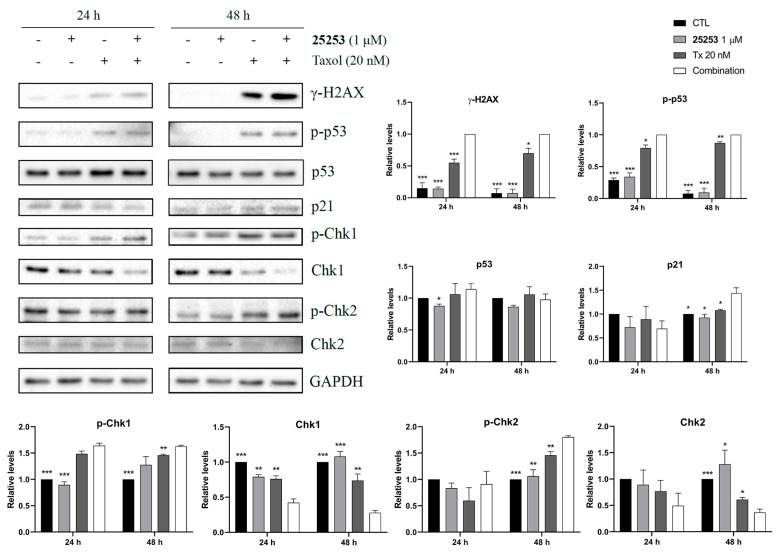
Induction of DNA damage by **25253** in combination with Taxol in ES-2 cells. ES-2 cells were treated with 1 μM **25253** and 20 nM Taxol either alone or in combination for 24 and 48 h and then harvested for Western blot analysis of DNA damage-related proteins. Quantitative data are presented as mean ± SEM of at least three independent experiments. Since γ-H2AX levels in CTL groups were extremely low, the levels in the combination groups were set as 1 for quantitative purpose. * *p* < 0.05, ** *p* < 0.01, *** *p* < 0.001 vs. combination.

It has been reported that Taxol induces DNA damage after prolonged mitotic arrest and mitotic slippage [17]. Western blot analysis of DNA damage-related proteins was, then, conducted to determine whether **25253** could enhance the effect of Taxol. As demonstrated in Figure 6, **25253** enhanced the induction of a DNA damage marker, γ-H2AX, by Taxol in ES-2 cells after 24 and 48 h of treatment. Interestingly, the induction of γ-H2AX was low but apparent at 24 h, and the signal was significantly increased at 48 h, suggesting the accumulation of DNA damage. Checkpoint kinases such as Chk1 and Chk2 can be activated by phosphorylation after DNA double-strand breaks and transmit DNA damage signaling to p53 [18]. Phosphorylated Chk1 (p-Chk1) levels were upregulated and total Chk1 protein levels were downregulated by Taxol and the combination of Taxol and **25253** at both 24 h and 48 h. A similar trend in Chk2 was observed at 48 h. p53 was markedly phosphorylated at Ser15 (p-p53^Ser15^) by Taxol and the combination of Taxol and **25253** at both 24 h and 48 h. Of note, **25253** significantly enhanced p-p53^Ser15^ levels induced by Taxol, indicative of further p53 activation, which led to clear induction of its downstream effector p21 at 48 h. Taken together, the enhancement of DNA damage was evident at 24 h and further increased after 48 h of treatment. Additionally, the downstream signaling was activated, leading to p53 activation and, eventually, apoptosis. This DNA damaging effect may also contribute to the synergistic induction of apoptosis by **25253** and Taxol.

### 2.6. **25253** Enhances the Inhibitory Effects of Taxol on Cell Migration and Invasion in ES-2 Cells

**Figure 7:**
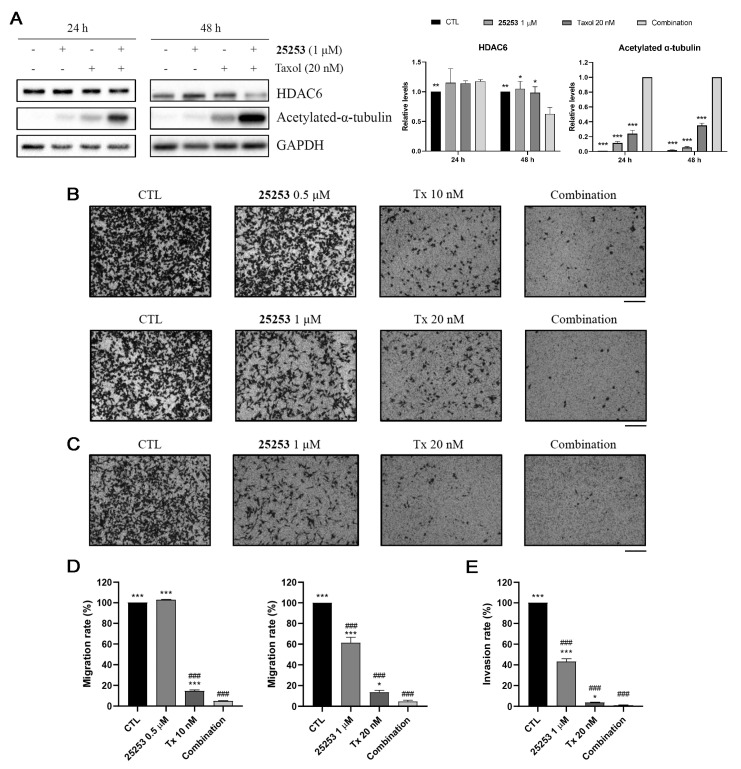
Compound **25253** enhances the inhibitory effect of Taxol on cell migration and invasion in ES-2 cells. (**A**) Compound **25253** and Taxol synergistically downregulated HADC6 and induced acetylated α-tubulin. (**B**) Compound **25253** potentiated the inhibitory effect of Taxol on cell migration. (**C**) Compound **25253** potentiated the inhibitory effect of Taxol on cell invasion. (**D**) Quantitative data of cell migration. (**E**) Quantitative data of cell invasion. Transwell cell migration and invasion assays were conducted for 24 h. Data are presented as mean ± SEM of at least three independent experiments. * *p* < 0.05, ** *p* < 0.01, *** *p* < 0.001 vs. combination. ^###^ *p* < 0.001 vs. CTL. Cell migration and invasion images were captured at a magnification of 100×. Scale bar: 200 μM.

α-Tubulin is one of the HDAC6 substrates [9] and Taxol itself can induce α-tubulin acetylation, which is associated with enhanced microtubule stability [19]. Western blot analysis was conducted to determine the combinatorial effect of **25253** and Taxol on the acetylation of α-tubulin. As demonstrated in Figure 7A, **25253** and Taxol synergistically enhanced the acetylation of α-tubulin at 24 h and this effect persisted at 48 h. Moreover, HDAC6 was significantly downregulated at 48 h, which may also partly explain the increased acetylation of α-tubulin.

Since the acetylation status of α-tubulin plays a crucial role in microtubule dynamics, which may impact cell mobility [20], a transwell cell migration assay was conducted to determine the effect of **25253** and Taxol on cell migration. As shown in Figure 7B,D, ES-2 cells treated with 0.5 μM **25253** for 24 h exhibited similar cell migration (102.88 ± 0.34%) compared to the control (100%), while 1 μM **25253** exerted a moderate inhibitory effect (61.32 ± 5.37% cell migration). Both 10 nM and 20 nM Taxol showed strong inhibitory effects, with cell migration of 14.62 ± 1.4% and 13.56 ± 1.89%, respectively. As for the combination treatments, 0.5 μM **25253**/10 nM Tx and 1 μM **25253**/20 nM Tx suppressed cell migration down to 4.92 ± 0.34% and 4.57 ± 1.31%, respectively, which was statistically significant compared to single treatments with Taxol (Figure 7D). The results indicated that **25253** significantly enhanced the inhibitory effect of Taxol on cell migration.

Other than cell migration, cell invasion is also a crucial hallmark of cancer metastasis. A transwell cell invasion assay was then performed to examine whether the combination of **25253** and Taxol also inhibited cell invasion. As demonstrated in Figure 7C,E, 1 μM **25253** exerted a moderate inhibitory effect (43.37 ± 2.58% relative cell invasion), while 20 nM Taxol exhibited a strong inhibitory effect (3.6 ± 0.48% relative cell invasion) on the invasion of ES-2 cells. The combination of 1 μM **25253** and 20 nM Taxol further inhibited cell invasion down to 1.1 ± 0.48% which was statistically significant compared to Taxol alone (*p* < 0.05) (Figure 7E). These results indicated that the combination of **25253** and Taxol not only exhibited enhanced the inhibition of cell migration but also significantly suppressed cell invasion; therefore, it may be a very effective strategy against cancer metastasis.

## 3. Discussion

The first-line treatment for ovarian cancer typically involves surgical intervention aiming at debulking the tumor, followed by chemotherapy with taxanes or platinum-based drugs to eliminate any remaining cancer cells [4,5]. However, the low overall survival rate and poor prognosis make the finding of a new treatment strategy an urgent need. Studies have found elevated HDAC6 in ovarian cancer, which helps the cancer cell to develop chemoresistance and possible metastasis such as anchorage-independent proliferation, cell migration, and invasion [21,22]. Therefore, targeting HDAC6 presents a promising therapeutic approach against ovarian cancer. Furthermore, it has been reported that HDAC6 inhibitors such as ACY-241 (citarinostat) [19,23] and A452 [23] enhance the activity of paclitaxel in ovarian cancer cells.

To identify novel potent HDAC6 inhibitors against ovarian cancer, the anticancer activity of 46 potential HDAC6 inhibitors was evaluated using the MTT assay in ES-2 ovarian cancer cells. The structures of these 46 compounds are illustrated in Figure S1 and their antiproliferative effects in ES-2 cells are shown in Figure 1A. In general, quinazolinone-based derivatives with a styryl linker to connect the zinc-binding group exhibited more potent antiproliferative effects than those with a benzyl linker. Three most potent candidates, **25253** (#16), **25276** (#14), and **25278** (#17), were identified with IC_50_ values of 1.19 μM, 1.17 μM, and 1.53 μM, respectively (Figure 1C). The representative candidate **25253** displayed the ability of long-term growth inhibition revealed by the colony formation assay (Figure 1E,F) and selective inhibition of HDAC6 by Western blot analysis of acetylated α-tubulin and histone H3 (Figure 1G). The inhibitory effects of **25253**, **25276**, and **25278** on the enzyme activities of purified HDAC1, HDAC6, HDAC8, and HDAC11 were also determined as shown in Table S2, confirming the HDAC6 selectivity. Although **25253** only showed an 8.67-fold HDAC6 selectivity over HDAC1 based on the enzyme activity assay, the selectivity inside the cell seemed to be better, as revealed by its effects on the acetylation of α-tubulin and histone H3 (Figure 1G). Intriguingly, **25276** and **25278** displayed similar antiproliferative effects compared to **25253** in ES-2 cells (Figure 1A–C) in spite of better inhibitory effects toward purified HDAC6 (Table S2). It remains a possibility that these HDAC6 inhibitors may exert their anticancer activity in part via the inhibition of other HDACs, and further investigation is needed to clarify the issue.

The IC_50_ of **25253** was 0.61 μM in TOV21G cells (MTT assay after 72 h treatment) (Table S1), likely to be more potent than ACY-241 (IC_50_ of 6.1 μM, MTS assay after 72 h treatment) [19] or equivalent to A452 (IC_50_ of 0.8 μM, CCK-8 assay after 48 h treatment) [23]. Furthermore, **25253** was much less toxic to normal human ovarian surface epithelial cells [24], with an IC_50_ value of 18.80 μM, and normal human dermal fibroblast cells, with an IC_50_ value of 17.85 μM (Figure S2).

To determine whether our HDAC6 inhibitors synergized with anticancer agents, **25278**, one of the most potent candidate compounds, was used for preliminary testing in combination with cisplatin, bortezomib, Taxol, and MK-2206 in ES-2 cells. The results indicated that Taxol had the best combinatorial effect with **25278** (Figure 2A). Some studies have reported that HDAC6 inhibitors can enhance the activity of Taxol in ARID1A-null ovarian cancer cells [23] and tumor models [19], partly due to the disruption of microtubule dynamics. In our study, we combined Taxol with the three candidates, **25253**, **25276**, and **25278**, and discovered that **25253** exhibited the strongest combinatorial effect with Taxol (Figure 2B). After a series of optimizations, we found that **25253** and Taxol with a molar ratio of 1000:20 exerted the best synergistic effect in ES-2 cells (Figure 2C) which express wild-type ARID1A protein [25], while a ratio of 1000:10 also exerted a synergistic effect in ARID1A-null TOV21G cells [23] (Figure 3A). The combination of **25253** and Taxol also significantly suppressed colony formation in long-term assays in ES-2 cells, indicating a sustained anticancer effect (Figure 2D). Taken together, our novel selective HDAC6 inhibitor **25253** demonstrated a synergic effect when combined with Taxol not only in ARID1A-null TOV21G cells but also in ARID1A wild-type ES-2 ovarian cancer cells.

Taxol alone and in combination with **25253** significantly disrupted the cell cycle distribution in ES-2 cells, most notably causing an increase in the subG1 population as early as 24 h (Figure 4A,B). Several studies have demonstrated that low concentrations of Taxol, as used in this study (5 or 10 nM), induce prolonged mitosis, leading to abnormal division and aneuploidy. These aneuploid cells may not progress through another cell cycle but may survive temporarily before progressing to apoptosis [15,16]. Thus, the subG1 cells detected at 24 h may be viable G1 rather than apoptotic cells. Indeed, Western blot analysis demonstrated the presence of cleaved PARP, an apoptosis marker, after 48 h of Taxol treatment but not 24 h, suggesting that subG1 cells were still alive at 24 h before eventually undergoing apoptosis at 48 h. The combination with **25253** significantly enhanced this effect of Taxol (Figure 5C).

Previous studies demonstrated that Taxol induced apoptosis through the intrinsic pathway by regulating Bcl-2 family proteins and caspase cascade [26,27]. Pro-survival proteins Bcl-2 and Bcl-XL were downregulated, and pro-apoptotic proteins Bax and Bak were increased, indicating that apoptosis induced by the combination of **25253** and Taxol was mediated through the intrinsic pathway. Changes in the Bcl-2 family proteins were apparent at 48 h, which was correlated with the induction of cleaved PARP, confirming the activation of apoptosis (Figure 5). Thus, the combination of **25253** and Taxol induced apoptosis via the intrinsic pathway in ES-2 cells, suggesting that this treatment could effectively cause ovarian cancer cell death.

Taxol induced DNA damage [17], and our study revealed that **25253** further enhanced this effect in ES-2 cells. As shown in Figure 6, Taxol alone slightly induced the DNA damage marker γ-H2AX at 24 h, which could be associated with partial activation of apoptosis but not full apoptosis [17]. A dramatic increase in γ-H2AX was induced by Taxol at 48 h, indicating the accumulation of DNA damage over time (Figure 6), which could be correlated with apoptosis (Figure 5C). The combination with **25253** further enhanced the DNA damaging effect of Taxol, resulting in even more pronounced increases in γ-H2AX (Figure 6). In response to DNA damage, both Chk1 and Chk2 were phosphorylated and activated, leading to the phosphorylation of p53 and induction of p21 (Figure 6), which may lead to cell cycle arrest (Figure 4) and, eventually, trigger apoptosis (Figure 5). In summary, the combination of **25253** and Taxol significantly induced DNA damage in ES-2 cells, which may, in turn, lead to activation of p53, upregulation of p21, cell cycle arrest, and, ultimately, apoptosis.

Cancer metastasis is typically considered the final stage of cancer progression and the cause of cancer death. Cell migration and invasion are key processes in cancer metastasis. It is well-known that Taxol stabilizes microtubules and blocks cell division [16], while HDAC6 inhibitors prevent HDAC6 from deacetylating cytoplasmic substrates including α-tubulin, a major component of microtubules [9]. Both Taxol and HDAC6 inhibitors promote the acetylation of α-tubulin, leading to hyperacetylation when combined [19,23]. Western blot analysis indicated that the combination of **25253** and Taxol significantly increased acetylated α-tubulin levels in ES-2 cells. Notably, HDAC6 was slightly downregulated by the combination treatment, which may also contribute to the higher acetylated α-tubulin levels compared to single treatments (Figure 7A). The hyperacetylation of α-tubulin disrupts microtubule dynamics, which are crucial for cell mobility. Indeed, transwell cell migration and invasion assays demonstrated that the combination of **25253** and Taxol suppressed cell migration and invasion in ES-2 cells more effectively than either single treatment (Figure 7B–E). The combination of **25253** and Taxol not only synergistically inhibits the growth but may also suppress the metastasis of ovarian cancer and, thus, may have profound clinical implications. Epithelial–mesenchymal transition (EMT) plays an important role in cancer metastasis, and further investigation is required to determine whether the combination of **25253** and Taxol inhibits cell mobility by reversing the EMT process.

In recent years, many novel therapies have been developed and tested in clinical trials for ovarian cancer. Targeted therapies such as PARP inhibitors, bevacizumab, and mirvetuximab, as well as antibody-drug conjugates and immunotherapies, have provided better survival rates and promising outcomes in clinical trials [28]. These advancements significantly change the traditional treatment landscape and offer new strategies for combating ovarian cancer. HDAC6 has recently emerged as a promising target for ovarian cancer treatment since it has been found to overexpress in ovarian cancer and is also involved in vital cancer progression processes. This study has provided evidence to support that the combination of selective HDAC6 inhibitors and Taxol may be a promising treatment strategy for ovarian cancer and encourages further investigation.

## 4. Materials and Methods

### 4.1. Chemicals

Tubastatin A, paclitaxel, cisplatin, PI, and crystal violet were obtained from Sigma (St. Louis, MO, USA). Bortezomib was purchased from AK Scientific (Union City, CA, USA). MK-2206 was purchased from BioVision (Mountain View, CA, USA). MTT was obtained from Invitrogen Life Technologies (Carlsbad, CA, USA). Stock solutions of HDAC6 inhibitors, SAHA (in-house synthesized), tubastatin A, paclitaxel, bortezomib, and MK-2206 were prepared in DMSO. Cisplatin and MTT were dissolved in PBS. Crystal violet was dissolved in 20% methanol.

### 4.2. Cell Lines and Cell Culture

ES-2 (CRL-1978, p63 from ATCC, Manassas, VA, USA) and TOV21G (BCRC 60407, p44 from Bioresource Collection and Research Center, Hsinchu, Taiwan) ovarian cancer cell lines were cultured in McCoy’s 5A medium supplemented with 10% fetal bovine serum (FBS), 1.5 mM L-glutamine, and antibiotics (100 units/mL penicillin, 100 μg/mL streptomycin, and 0.25 μg/mL amphotericin B). Cells were cultured at 37 °C in a humidified 5% CO_2_ atmosphere.

### 4.3. Cell Viability and Drug Combination Analysis

Cell viability was determined by the MTT assay. ES-2 and TOV21G cells (~3000 cells/well) were seeded into 96-well plates overnight and subjected to drug treatment for 72 h followed by the MTT assay as described previously [12]. The efficacy of drug combination was evaluated using two methods. The synergy score was calculated using SynergyFinder 3.0 software [13] and combination index was calculated from cell viability data using CompuSyn software [14].

### 4.4. Colony Formation Assay

ES-2 cells (250 cells/well) were plated into 12-well plates overnight and treated with **25253** and/or Taxol for 24 h and then cultured in drug-free medium for an additional 9 days to allow colony formation. Colonies were rinsed with PBS and stained with crystal violate (0.4% crystal violet in 20% methanol) for 20 min then rinsed with ddH2O and air-dried. Colonies with at least 50 cells were counted under the microscope and images were captured using the ChemiDoc MP Imaging System (Bio-Rad Laboratories, Hercules, CA, USA).

### 4.5. Western Blot Analysis

ES-2 cells were seeded into 6-well plates at 2.5 × 10^5^ cells/well or 1 × 10^5^ cells/well and treated with DMSO as the control or indicated compounds for 24 h or 48 h, respectively. Cells were harvested after treatment and lysed with RIPA buffer, and samples with 20–30 μg protein were subjected to SDS-PAGE and Western blot analysis as previously described [29]. Primary antibodies used included HDAC6, Bcl-XL, Bax, Bak, phophorylated Chk1 at Ser345 (p-Chk1), phophorylated Chk2 at Thr68 (p-Chk2), p-p53^Ser15^ (purchased from Cell Signaling Technology, Boston, MA, USA), Bcl-2 (Dako, Carpinteria, CA, USA), γ-H2AX (Millipore, Billerica, MA, USA), acetylated α-tubulin, Chk1, Chk2, cyclin D1, (Santa Cruz Biotechnology, Santa Cruz, CA, USA), acetylated histone H3, GAPDH, PARP (GeneTex, Irvine, CA, USA), hypophosphorylated RB (Hypo RB), RB (BD Biosciences, San Jose, CA, USA), and p53 and p21 (Sigma). Secondary antibodies used were HRP-conjugated anti-mouse and anti-rabbit IgG (Cell Signaling Technology). GAPDH was used as a loading control.

### 4.6. PI Staining and Flow Cytometric Analysis

ES-2 or TOV21G cells were plated into 12-well plates at 0.8–1.2 × 10^5^ cells/well, treated as indicated for 24 or 48 h, then harvested by trypsinization. Cells were fixed with 70% (*v*/*v*) ethanol at −20 °C for at least 30 min, centrifuged, and incubated in PI staining solution for 15–30 min in the dark. Flow cytometric analysis was conducted using FACSCalibur (BD Biosciences) and data were analyzed by FlowJo software v10 (Tree Star Inc., Ashland, OR, USA).

### 4.7. Annexin V-FITC/PI Double Analysis

ES-2 cells (2.5 × 10^4^ cells/well) were seeded into 12-well plates overnight and treated as indicated for 72 h. Cells were harvested by trypsinization and resuspended in 1× Annexin V-FITC binding buffer. Cells were then stained with Annexin V-FITC and PI using the Annexin V Apoptosis Detection kit (Santa Cruz Biotechnology) according to the manufacturer’s instructions. Cells were stained for 15 min in the dark and subjected to two-dimensional flow cytometric analysis using FACSCalibur. Acquired data were then analyzed by FlowJo software.

### 4.8. Transwell Cell Migration and Invasion Assays

Transwell inserts for 24-well plates with a polycarbonate membrane (8 μm pore size, Corning Inc., Costar, Kennebunk, ME, USA) were employed for both migration and invasion assays. For migration assay, ES-2 cells (3 × 10^4^ cells/insert) in serum-free medium with the indicated compounds were seeded onto the transwell inserts. For the invasion assay, the inserts were coated with 100 μL of diluted Matrigel matrix (Corning Inc., Bedford, MA, USA, protein concentration: 8.4 mg/mL). Matrigel was diluted with serum-free medium to a protein concentration of 300 μg/mL and coated onto the inserts at 37 °C for at least 60 min to allow for solidification. After removing the supernatant, 200 μL of cell suspension was seeded onto the Matrigel. The lower chamber was filled with 600 μL of drug-containing medium with 10% FBS. Following a 24 h incubation period, cells were fixed with 100% cold methanol for 10 min, stained with 0.4% crystal violet in 20% methanol for 20 min, and washed once with dH2O. Non-migratory or non-invasive cells were removed with cotton swabs, while migratory or invasive cells attached to the lower surface of the membrane were photographed. Migration or invasion was quantified using Image J 1.49v software (National Institute of Health, Bethesda, MD, USA), and data were presented as the percentage relative to the DMSO vehicle control.

### 4.9. Data Analysis

Quantitative data are presented as mean ± SEM of at least three independent experiments except for the preliminary drug combination test. Statistical significance was assessed using two-sided Student’s *t*-test and *p* < 0.05 was considered statistically significant.

## Data Availability

Data presented in the study are included in the article/Supplementary Materials, further inquiries can be directed to the corresponding authors.

## Supplementary Materials

The following supporting information can be downloaded at: https://www.mdpi.com/article/10.3390/molecules30132793/s1, Table S1: IC_50_ values of **25253** and Taxol in single or combination treatments in ES-2 (molar ratio 1000:20) and TOV21G (molar ratio 1000:10) ovarian cancer cells; Table S2: The inhibitory effects of **25253**, **25276** and **25278** on the enzyme activities of HDACs; Figure S1: Structures of 46 potential novel HDAC6 inhibitors; Figure S2: Dose-response curves of **25253** in normal human ovarian surface epithelial cells and normal human dermal fibroblast cells.

## Abbreviations

The following abbreviations are used in this manuscript:

PARP	poly (ADP-ribose) polymerase
HDAC	Human histone deacetylase
SAHA	Suberoylanilide hydroxamic acid
IC_50_	50% inhibitory concentration
TST	Tubastatin A
SC	Synergy score
CI	Combination index
CI_50_	Combination index at IC_50_
PI	Propidium iodide
Tx	Taxol
