# Supplementary material for: A Novel HDAC6 Inhibitor Enhances the Efficacy of Paclitaxel Against Ovarian Cancer Cells
Source: Molecules. 2025 Jun 28;30(13):2793. doi: 10.3390/molecules30132793 (PMC12251222; doi:10.3390/molecules30132793)
Supplement: Supplementary file 1 [file molecules-30-02793-s001.zip › molecules-3719168-supplementary.pdf]

Table S1. IC_{50} values of **25253** and Taxol in single or combination treatments in ES-2 (molar ratio 1000:20) and TOV21G (molar ratio 1000:10) ovarian cancer cells.

**IC_{50}**[1]	**25253 (μM)**[S]	**Tx (nM)**[S]	**25253 (μM)**[C]	**Tx (nM)**[C]
**ES-2**	1.13	17.41	0.43	8.64
**TOV21G**	0.61	7.19	0.288	2.88

[1] Cells were treated for 72 h and IC_{50} values were calculated by CompuSyn software based on cell viability measured by the MTT assay from three independent experiments. [S] single treatment. [C] combination treatment.

Table S2. The inhibitory effects of **25253**, **25276** and **25278** on the enzyme activities of HDACs.

**Cpds, IC_{50} (μM)**[1]	**HDAC1**	**HDAC8**	**HDAC11**	**HDAC6**
**25253**	4.94 (8.67)[2]	3.15 (5.53)	1.80 (3.16)	0.57
**25276**	4.37 (43.7)	3.43 (34.3)	5.15 (51.5)	0.10
**25278**	5.33 (35.5)	3.64 (24.3)	6.56 (43.7)	0.15

[1] Compounds (Cpds) were submitted to Reaction Biology Corporation for enzyme inhibition assays against human HDACs and tested in 10-dose IC_{50} mode in singlet with 3-fold serial dilution starting from 30 μM. HDACs are grouped into four classes. HDAC1 and HDAC8 belong to class I, HDAC6 is a class IIb histone deacetylase, and HDAC11 is a class IV histone deacetylase. **25253**, **25276** and **25278** exhibited selective HDAC6 inhibitory effects.
[2] The IC_{50} ratios of HDACs and HDAC6 are indicated in the parentheses.

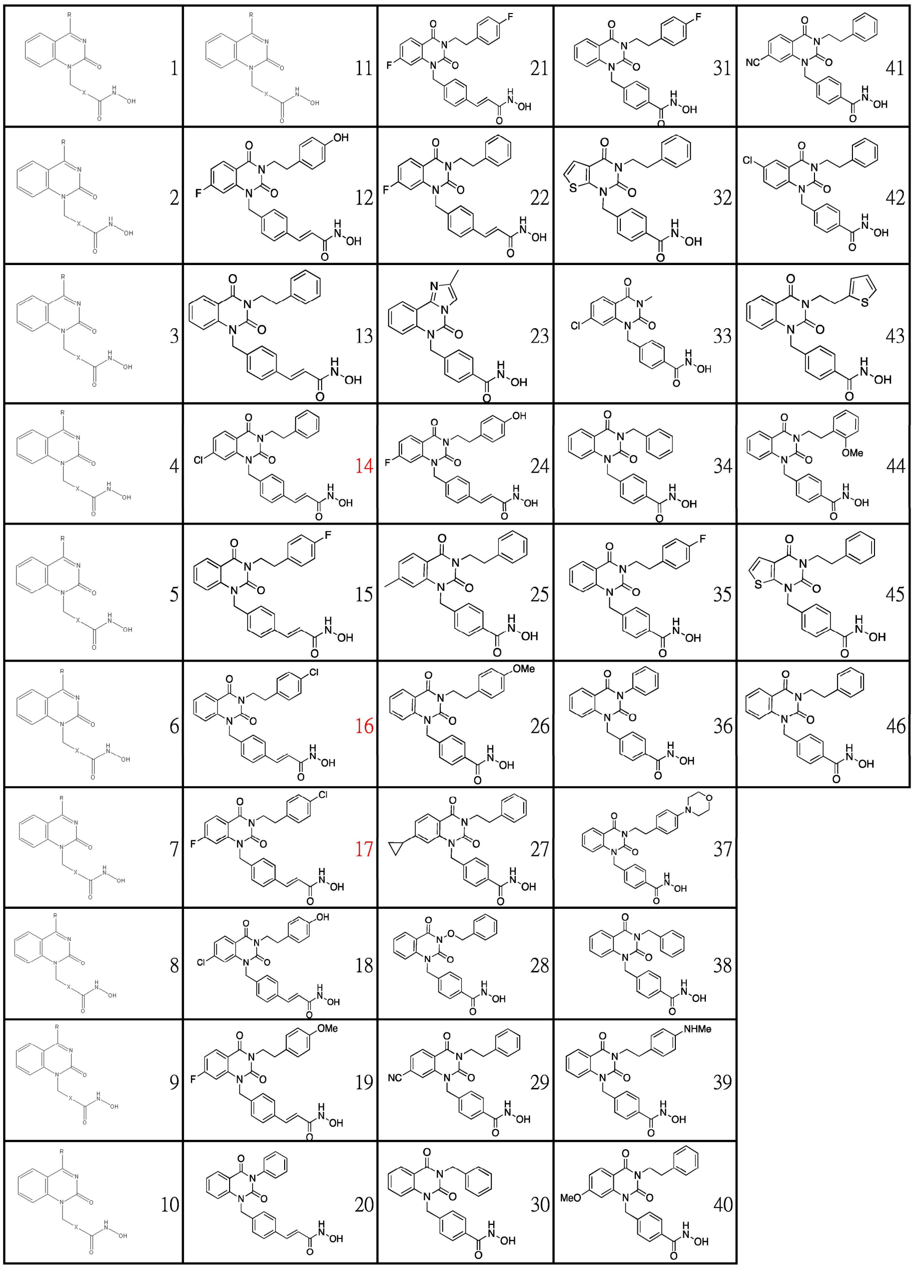

Figure S1. Structures of 46 potential novel HDAC6 inhibitors. Three compounds with the most potent antiproliferative activities are highlighted in red. Compounds 1-11 are 4-arylquinazolinone derivatives under patent preparation with R as aryl or heteroaryl groups and X as aryl, alkenyl or heteroaryl groups.

A **B**

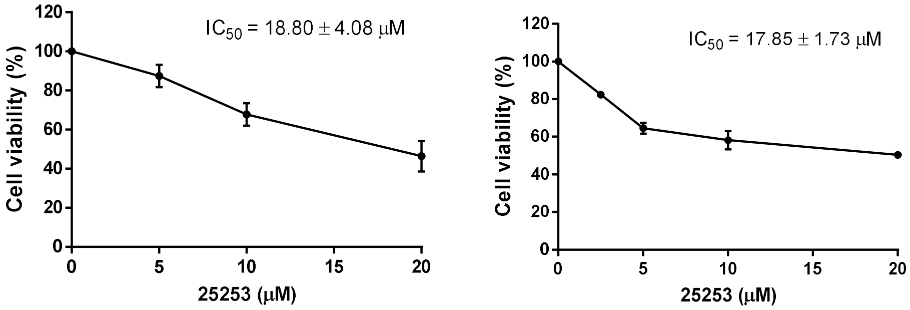

Figure S2. Dose-response curves of **25253** in normal human ovarian surface epithelial cells and normal human dermal fibroblast cells. Cells were seeded in 96-well plates, treated with **25253** for 72 h, and cell viability was measured by the MTT assay. (A) Primary normal human ovarian surface epithelial cells were cultured in MCDB105/medium199 supplemented with 15% FBS and antibiotics. Approximately 4000 cells were seeded to each 96-well for treatment with **25253**. Data are presented as mean ± SEM of three independent experiments. (B) Primary normal human dermal fibroblast cells (C-12302, PromoCell) were cultured in PromoCell Fibroblast Growth Medium (C-23020) according to the manufacturer's instructions. Approximately 3000 cells were seeded to each 96-well for treatment with **25253**. Data are presented as mean ± SEM of two independent experiments.
